# Beneficial Effects of Purified Cytoplasm of Pollen Associated with Vitamin D and Vitamin K2 on Menopausal Symptoms and Bone Health

**DOI:** 10.3390/nu18162734

**Published:** 2026-08-21

**Authors:** Anna Capozzi, Anna Fagotti, Davide Sacco, Anna Pasi, Rossella Mellino, Stefano Lello

**Affiliations:** 1 Department of Women, Child and Public Health, Fondazione Policlinico Universitario Agostino Gemelli IRCCS, 00168 Rome, Italy; anna.fagotti@policlinicogemelli.it (A.F.); lello.stefano@gmail.com (S.L.); 2 Dipartimento Interdisciplinare di Medicina (DIM), Università Degli Studi di Bari Aldo Moro, 70126 Bari, Italy; 3 Polistudio Donna si Cura, 48124 Ravenna, Italy; apasiravenna@gmail.com; 4 Poliambulatorio Polispecialistico Domus Mulieris, 01100 Viterbo, Italy; rossellamellinoprivato@gmail.com

**Keywords:** Purified Cytoplasm of Pollen, menopause, perimenopause, hot flashes, mood disorders, sleep, quality of life

## Abstract

**Background:** The menopausal transition may be accompanied by several disabling symptoms which impair quality of life. Many women cannot proceed with and/or refuse hormonal replacement therapy (HRT) and, thus, they rely on non-hormonal remedies to manage climacteric syndrome. **Methods:** This was a retrospective study analyzing the impact of a combination of Purified Cytoplasm of Pollen (PCP) with vitamin D (vitD) and vitamin K2 (vitK2) on menopausal symptoms and bone parameters in a sample of symptomatic women (Group A, *n* = 22) compared to controls (Group B, *n* = 23). **Results:** We found that blood levels of vitD (25OHD) significantly increased within Group A (*p* = 0.0004) whereas no significance was reached within Group B (*p* > 0.05) after a three-month therapy. Compared with the baseline, serum *C*-terminal telopeptide of type I collagen (CTX) turned out to be significantly reduced within Group A (*p* = 0.0415) but not within Group B (*p* > 0.05). Comparing Group A and Group B, the values of CTX significantly differed after treatment (*p* = 0.0458). In comparison to the baseline, significant differences emerged within Group A for physical, psychosocial, and vasomotor domains of Menopause-Specific Quality of Life (MenQol) (*p* < 0.05) whereas no significant difference was found for the sexual domain (*p* > 0.05). Comparing Group A and Group B, significant differences were observed for all MenQol domains (*p* < 0.05), except for the sexual domain (*p* > 0.05) after therapy. **Conclusions:** Based on our findings, the combination of PCP, vitD, and vitK2 could be used for managing vasomotor and mood disturbances associated with menopause, whilst also offering the added benefit of maintaining bone health.

## 1. Introduction

Hot flashes (HFs) are the most frequent and bothersome symptoms of perimenopause, along with sleep disorders and mood fluctuations [1]. It has been reported that HFs could be related to impaired bone health from premenopause, facilitating bone deterioration and, thus, generally rendering symptomatic women more prone to osteopenia or osteoporosis [2].

Hormone replacement therapy (HRT) is regarded as the main treatment for alleviating climacteric syndrome and a primary prevention measure for many chronic conditions, such as cardiovascular diseases and osteoporosis [3]. However, many women cannot receive and/or decline hormone therapy or may be affected by menopausal symptoms even outside the time period considered the “window of opportunity” for initiating HRT, that is, before sixty years of age and/or within ten years after the last menstrual period, at which point the risk–benefit profile becomes unfavorable for the initiation of HRT [4].

In this context, several non-hormonal therapies have been proposed to reduce the principal symptoms of menopause [5,6].

Purified Cytoplasm of Pollen (PCP) is a non-hormonal treatment that has been shown to be effective and safe in women with menopause including those with a history of cancer, since it can reduce HFs as well as sleep and mood disorders, globally improving quality of life [7]. Notably, several supplements like vitamin D (vitD), calcium, and vitamin K2 (vitK2) could prevent bone loss, potentially enhancing the beneficial effects of specific compounds on bone health and/or improving bone health independently [8]. A large amount of evidence has highlighted that hypovitaminosis D is a highly prevalent pathological condition worldwide and recent data has shown that dietary intake of vitamin D is particularly low among women [9,10].

The objective of this retrospective observational study is to evaluate the efficacy of a combination therapy consisting of PCP associated with vitD and vitK2 on menopausal symptoms and the main parameters of bone metabolism.

## 2. Materials and Methods

This was a retrospective observational study on a sample of pre- and post-menopausal women affected by climacteric symptoms and treated with PCP associated with vitD and vitK2 for at least three months and compared with a group of untreated women.

The study involved patients referred to different gynecological clinics in Italy for menopausal syndrome from May 2025 to November 2025 and followed up with for at least three months. More specifically, we evaluated data previously collected in the databases of the gynecological clinics that participated in the study (Figure S1).

Our retrospective evaluation included women with the following characteristics:

- Being an Italian woman aged between 45 and 60 years;
- Being in postmenopause according to the established clinical criteria (last menstrual cycle 12 months ago or a period of amenorrhea ≥ 6 months associated with a blood assessment of Follicle-Stimulating Hormone (FSH) > 40 UI/L and Estradiol < 20 pg/mL before the basal evaluation) or being in premenopause with irregular menses (menstrual cycles shorter than 21 days or longer than 36 days);
- Having at least three episodes of HFs at baseline;
- Being available to give their written consent to the collection of their clinical data.

We excluded women who were younger than 45 and older than 60 years of age, those with ongoing regular menstruation, and/or those with less than three HFs. Other exclusion criteria included being on treatment with HRT, anti-depressants, antihypertensives, and/or supplements like vitD and its analogues, calcium, and/or vitK2 or other nutraceuticals. We did not include in our evaluation patients who had been previously affected by sleep and/or mood disorders, before the menopausal period.

Patients were divided into two main groups according to their choice, based on medical advice, to take PCP as a single oral formulation (Femal Osteo), containing 320 mg PCP, 25 mcg vitamin D3 (cholecalciferol), 10 mg vitamin E (tocopherol), 180 mcg vitamin K2 (menaquinone 7-MK7), and 1,4 mg vitamin B6 (Group A), or to refuse any pharmacological treatment for vasomotor symptoms (VMS) (Group B).

The assessment of the following serum parameters was performed at baseline (T0) and after three months of observation (T3) for both groups: follicle-stimulating hormone (FSH), luteinizing hormone (LH), estradiol (E2), vitamin D (25OHD), parathyroid hormone (PTH), calcium (Ca), and phosphorus (P). Two markers of bone turnover (BTMs) were evaluated in blood: *C*-terminal telopeptide of type I collagen (CTx) for bone resorption and Osteocalcin (OC) for bone formation.

Hormone levels and BTMs were assessed through electrochemiluminescent immunoassay (ECLIA), whereas Ca and P were measured by atomic absorption spectrophotometry. Serum total 25OHD levels were measured using automated Abbott Architect 25OHD immunoassay (bias ng/mL% = +0.4/1.7–4.7 between 20 and 40 ng/mL according to Vitamin D External Quality Assurance Scheme). Vitamin D sufficiency was defined as serum levels of 25(OH)D ranging between 30 and 100 ng/mL, according to the US Endocrine Society Clinical Practice Guidelines [11]. We collected anthropometric parameters (i.e., age, height, weight, and BMI). More specifically, height and weight were measured using a digital scale with Seca 769 (Seca, Hammer Steindamm 3-25, Hamburg, Germany) whilst BMI was calculated by dividing body weight by height squared (kg/m^2^). Lumbar and femoral (total and neck) BMD (g/cm^2^) was assessed using DXA Horizon (Hologic Italia S.r.l.: Viale Città d’Europa 681, 00144 Rome, Italy). The TBS was calculated through anteroposterior scans of the spine at the L1–4 level using the TBS iNsight software, Version 3.1.2.

All women completed the Menopause-Specific Quality of Life (MENQOL) questionnaire before and after three months of treatment. It is a self-reported measure that specifically assesses QOL in menopause [12]. Respondents were asked whether they had experienced any of the 29 symptoms in the past month and to rate how bothersome each symptom was perceived according to a 7-point scale ranging from 0 (not at all bothered) to 6 (extremely bothered). The 29 items were combined into four main domains: vasomotor, psychological and social, physical, and sexual. For analysis, the questionnaire score was 1 for “no”, 2 for “yes”, and from 3 for “yes but not bothered” to 8 for “yes, extremely bothered”. The domain score was the mean of the converted item scores forming this domain and it ranged from 1 to 8 [12].

The primary objectives of our analysis were as follows:

- Assessing the efficacy of the compound taken by Group A compared to Group B in the management of menopausal symptoms evaluated through MENQOL;
- Evaluating the possible modifications of BTMs and calcium–phosphorus–PTH–25OHD balance within Group A and Group B after three months and the differences between groups;

The secondary outcomes of our study were as follows:

- Finding potential correlations between 25OHD and TBS;
- Analyzing the relationship between CTX and the severity of menopausal symptoms.

Differences between T0 and T3 of all investigated parameters were studied within the same group and between Group A and Group B.

To minimize potential sources of bias inherent to the retrospective observational study design, rigorous methodological strategies were employed for cohort selection and outcome measurement. The risk of selection bias was mitigated by applying strict inclusion and exclusion criteria; specifically, patients younger than 45 or older than 60 years of age, women with regular menstrual cycles, and those experiencing fewer than three hot flashes (HFs) per day were excluded. To isolate the true effect of the treatment and eliminate confounding factors, we excluded patients who had been taking PCP for less than 15 or more than 45 days, as well as those receiving potentially interacting medications (e.g., HRT, antidepressants, antihypertensives, and serotonin modulators), and women with sleep or mood disorders predating the menopausal period. The rigorous application of these criteria allowed us to isolate the effects of the combined therapy and establish two experimental cohorts (Group A and Group B) that exhibited no statistically significant differences at baseline regarding anthropometric parameters, hormonal profiles, and severity of symptoms (Table 1).

The study size was determined by the availability of patients who met all inclusion and exclusion criteria within the medical records of the participating centers during the observation period. A total of 45 patients were included in the final analysis. The cohort was divided into two groups: Group A (*n* = 22), consisting of patients who received the combined supplement (PCP, vitamin D3, and vitamin K2), and Group B (*n* = 23), which served as the control group. This sample size represented the total number of consecutive cases with complete clinical and biochemical data available for both the baseline (T0) and the three-month follow-up (T3).

### Statistical Analysis

Statistical analyses were performed using Python (version 3.14) and its data analysis libraries, including Pandas (Version 3.0.1) for data manipulation and SciPy.stats (Version 1.17.1) for statistical testing. The normality of data distribution for each variable was assessed using the Shapiro–Wilk test. Continuous variables were reported as median and interquartile range (IQR, 1st–3rd quartiles), as indicated in Table 1 and Table 2. MENQOL results were expressed as domain scores, calculated according to the standard scoring rules of the questionnaire, and treated as continuous variables for all statistical analyses.

To evaluate the comparability of the two groups at baseline (T0), the Mann–Whitney U test was applied, as the non-normal distribution of the data precluded the use of an independent *t*-test. To evaluate the efficacy of the intervention, the change from baseline to the three-month follow-up was calculated for each parameter (Δ = T0 − T3). The comparison of these deltas between Group A and Group B was performed using the Mann–Whitney U test. Additionally, within-group comparisons to assess changes from baseline to the three-month follow-up (T0 vs. T3) were performed using the Wilcoxon signed-rank test. To comprehensively account for multiple testing, raw *p*-values across all statistical tests performed were adjusted using the Two-Stage Benjamini–Krieger–Yekutieli False Discovery Rate (FDR) procedure and the magnitude of the effects was estimated using the rank-biserial correlation. Regarding missing data, only patients with a complete set of values for the primary and secondary outcomes were included in the final analysis. For all statistical tests, a *p*-value < 0.05 was considered statistically significant.

## 3. Results

A total of 45 patients were included in the final analysis of this study. The cohort was divided into two experimental groups: Group A (*n* = 22), consisting of patients who received the combined supplement of PCP, vitamin D3, and vitamin K2, and Group B (*n* = 23), which served as the control group. As demonstrated in Table 1, the two cohorts were homogeneous at baseline (T0), exhibiting no statistically significant differences across all investigated parameters. This lack of significance (*p* > 0.05) applied to all anthropometric data, serum and hormonal profiles, densitometric measurements, and baseline symptom severity scores, ensuring that the two groups were comparable prior to the intervention.

As for the value of PTH, following FDR correction, no significant changes were observed at T3 in comparison with T0 within either Group B or Group A, nor in the comparison of the treatment effect between Group A and Group B (all FDR-adjusted *p* > 0.05).

We found that serum Ca did not significantly change at T3 in comparison with T0 within both Group A and Group B, nor between the two groups (all FDR-adjusted *p* > 0.05).

With regard to the levels of *p*, after adjusting for multiple comparisons, no statistically significant differences were found in the comparison between T3 and T0 within Group B, nor between Group A and Group B (all FDR-adjusted *p* > 0.05). Similarly, no significant difference was found between T0 and T3 in Group A (FDR-adjusted *p* > 0.05).

**Figure 1:**
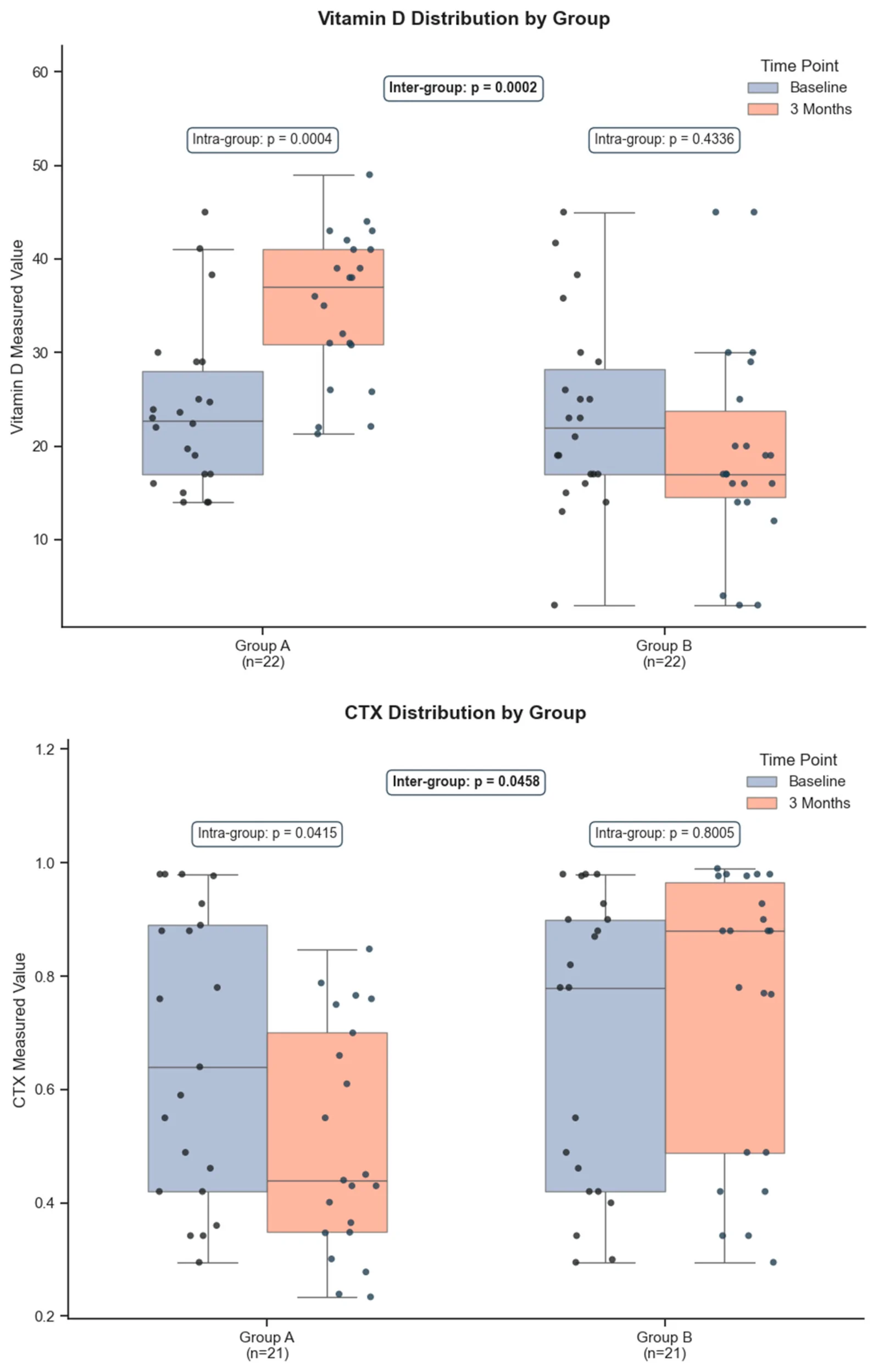
Distribution of serum vitamin D and *C*-terminal telopeptide (CTX) levels at baseline (T0) and after 3 months (T3) in Group A (treated) and Group B (untreated). Boxplots depict the median and interquartile ranges, with overlaid individual observations to provide a transparent representation of the data distribution given the sample size. The plots show that while Group A demonstrated a significant increase in Vitamin D, with median levels rising from 22.7 to 37.0 ng/mL, and a significant decrease in CTX levels from 0.64 to 0.44 ng/mL, Group B showed no statistically significant variations in either parameter over the 3-month observation period. These results illustrate that the combined therapy (Purified Cytoplasm of Pollen, vitamin D3, and vitamin K2) effectively optimized vitamin status and reduced bone resorption markers compared to the untreated control group.

Statistically significant differences were found for 25OHD between T0 and T3 in Group A, showing a +63.0% increase (*p* = 0.0004) and in the comparison between Group A and Group B at T3 (*p* = 0.0002), as shown in Figure 1. No significance was reached within Group B for the 25OHD analysis (*p* > 0.05).

The values of CTX were significantly reduced when comparing T3 with T0 within Group A, showing a −29.3% decrease (*p* = 0.0415) but not within Group B (*p* > 0.05). A statistically significant difference in CTX was observed in the comparison between Group A and Group B at T3 (*p* = 0.0458), as shown in Figure 1. No significant difference was observed in the evaluation of OC (*p* > 0.05).

As regards MenQOL questionnaire, HFs were significantly reduced within Group A at T3 (*p* = 0.0004) and in the comparison between Group A and Group B at T3 (*p* = 0.0001) but not within Group B (*p* > 0.05) (Appendix A).

Considering the domains of MenQol, we did not find statistically significant correlations between the domains and parameters of bone quality and/or density, such as lumbar and/or femoral T-score, BMD or TBS at T0 in the total sample except for the psychosocial domain (*p* = 0.02).

We found statistically significant differences between T0 and T3 within Group A for the physical, psychosocial, and vasomotor domains, with reductions of −40.3%, −50.0%, and −57.9%, respectively (*p* < 0.05), whereas no significant difference was found for the sexual domain (*p* > 0.05) (Table 2).

No differences were observed in any domain between T3 and T0 within Group B (*p* > 0.05).

Comparing Group A and Group B at T3, significant differences were found in all MenQol domains (*p* < 0.05), except for the sexual domain (*p* > 0.05).

The comparison of the median levels of 25OHD among women with normal TBS and low TBS revealed a statistically significant difference (*p* = 0.02).

We did not find a significant correlation between CTX levels and the severity of symptoms (*p* > 0.05).

## 4. Discussion

This retrospective study evaluated the effects of a combination of PCP with supplements influencing bone metabolism like vitD and vitamin K2 on both menopausal syndrome and bone health in a group of symptomatic menopausal women (Group A) in comparison to controls (Group B) after three months.

No significant changes were observed in PTH levels (*p* > 0.05). However, at T3, we found a statistically significant difference in phosphorus levels between Group A and Group B (*p* = 0.02), primarily driven by a reduction observed in Group B. Furthermore, no intra- or inter-group differences were found regarding Ca (*p* > 0.05).

These findings could be explained by the favorable influence of vitD supplementation on the maintenance of PTH-Ca-P homeostasis within Group A in comparison with Group B [13]. This aspect might influence, on one hand, bone health and, on the other hand, general well-being, considering the potential additional extra-skeletal benefits of vitD on women’s health [14]. As expected, 25OHD resulted in a significant increase in supplemented Group A (*p* = 0.00042) but not within Group B at T3 (*p* > 0.05). In the comparison between groups at T3, the difference reached statistical significance (*p* = 0.0002).

Interestingly, the comparison of median baseline 25OHD levels in the total sample of women showed a statistically significant difference (*p* = 0.01) in the comparison of subjects with normal TBS and those with low TBS. In other words, our results seem to suggest that an adequate bone quality, indicated by TBS within the normal range, may be associated with higher levels of 25OHD, confirming that vitD status may favorably impact bone density as well as bone microarchitecture [15,16].

As for BTMs, no significance was observed for OC (*p* < 0.05), likely due to the missing values of this BTM in data collection. However, we found a statistically significant difference of the values of CTX between T3 and T0 within Group A (*p* = 0.0415) but not within Group B (*p* > 0.05). Moreover, Group A and Group B differed significantly for CTX at T3 (*p* = 0.0458).

It is worth noting that a reduction in BTMs is an expected effect of drugs used to reduce bone turnover in case of bone fragility and may be predictive of a clinically favorable response to treatment [17,18]. Our results are noteworthy considering that the time of study observation is brief, and the administered compound is a combination of different supplements rather than a true anti-resorptive drug. We could hypothesize that the supplementation of vitD positively influenced bone turnover in Group A, contributing to the mitigation of bone resorption [19]. Additionally, the association with vitK2 may have further strengthened vitD activity [20]. It has been supposed that menaquinones are involved in the preservation of bone integrity not only through the well-known appropriate carboxylation of OC but also through a supposed modest inhibition of the pro-resorptive factor RANKL [21]. Therefore, although with caution, we may infer that vitD and vit K2 might act in a synergistic manner to mitigate bone turnover in our treated group.

At the same time, the benefits on HFs produced by PCP may have further reduced the potential negative effects of vasomotor symptoms on bone deterioration. A considerable body of evidence has demonstrated that HFs can affect bone health, showing that symptomatic women undergoing perimenopause usually have more marked bone loss in comparison to asymptomatic women [2]. Furthermore, several studies have reported that HFs as well as sleep disorders are mutually associated with several chronic diseases, like cardiovascular diseases and osteoporosis [22]. The increased levels of cortisol may have a key role in the etiopathogenesis of the latter clinical conditions, partially explaining the relationship between menopausal syndrome and health risks [23].

Therefore, all the components of the compound administered to Group A may have contributed to achieving overall well-being in treated subjects [22].

However, we did not find a significant correlation between CTX levels and the severity of symptoms (*p* > 0.05) or between the vasomotor domain of MenQol and T-score, BMD or TBS, probably owing to the limited sample size.

On the other side, our results seem to be consistent with a substantial body of literature that has demonstrated the benefits of PCP on menopausal syndrome in premenopause and postmenopause [24]. We found significant differences in the comparison between Group A and Group B after three months of treatment for vasomotor, psychosocial, and physical domains of MenQol (*p* < 0.05). A previous multicenter observational Italian study on 128 menopausal women reported significant differences for all items of the Greene Climacteric Scale, thus confirming the efficacy of PCP in treating climacteric syndrome [25]. Another study comparing a control group and another two groups respectively treated with isoflavones and PCP after three and six months showed that PCP can have a positive impact on HFs and sleep quality and showed greater efficacy compared to both controls and patients treated with isoflavones [26]. Those results could be explained by the fact that isoflavones may saturate estrogen receptors (ERs) and could be deeply subjected to individual intestinal metabolism, impairing the clinical response to treatment in the medium to long term. Conversely, PCP lacks any estrogenic activity: this property represents a crucial aspect to be taken into account to personalize therapy, as PCP may also be useful for treating symptomatic menopausal women that need to avoid any potential interaction with hormone receptors, like those affected by breast cancer (BC) [27]. Just in the latter case, a study has demonstrated that PCP is efficacious in the management of vasomotor symptoms but not in the treatment of sexual function, as reported by our study [28]. On the other hand, although the PCP may primarily act via serotoninergic pathways, it cannot overcome the complex mechanisms that modulate sexuality [28].

Notably, our analysis highlighted that treated women showed greater overall benefit compared with controls. These findings could be consistent with adjunctive mechanisms of action that have been recently described for PCP [29]. A study conducted in vitro by Appel et al. [29] reported that PCP can also act through GABAergic and dopaminergic pathways, potentially explaining the positive consequences of PCP treatment at different levels.

Many consequences of the therapy were unexpected, including improvements in skin quality and body weight compared with what we could observe for Group B. These findings may be explained by the overall benefits of PCP therapy on quality of life rather than as a potential estrogenic activity [30]. In fact, consistently, no significant difference regarding vaginal dryness between groups has been reported at T3 (*p* > 0.05).

To our knowledge, although retrospective, this is the first study reporting the efficacy of a compound composed of PCP, vitD, and vitK2 compared with controls. Our evaluation showed that this supplement could be useful not only to treat climacteric syndrome, but it could also help prevent bone deterioration associated with menopause, potentially counteracting increased bone turnover [31]. It should be noted that bone loss, as well as vasomotor symptoms, may begin many years before the final cessation of menstruation, when irregular menses may occur [32]. The availability of a supplement as an alternative to estroprogestins or anti-resorptive drugs that could globally help improve women’s quality of life may be extremely useful during the transitional period from pre- to postmenopause and even beyond, when HRT is not the treatment of choice.

A potential strength of our study could be the inclusion of a group of patients who refused to be treated with any supplement for their climacteric symptoms. This aspect may make our analysis more statistically robust, thereby potentially overcoming the limitations regarding the risk–benefit ratio evaluation emerging from other studies [33].

However, our study had some limitations:

- The analysis was conducted retrospectively and, thus, it was not designed as a randomized controlled trial that compared a treatment group with a placebo.
- The supplement taken by the treated group consists of various components; therefore, many compounds could be potentially implicated in its putative effects
- The sample analyzed was small.
- The data referred to a limited period of observation of three-months:; therefore, the findings could not be extended to a longer period, at least at present.
- Additionally, it could be advisable to consider more parameters of bone health to obtain more robust conclusions regarding the long-term use of this supplement. Anyway, these results are preliminary since our observation is ongoing.

## 5. Conclusions

In conclusion, our study seems to suggest the favorable impact of PCP on vasomotor and psychological symptoms of women in pre- and postmenopause and its potential role, when combined with vitD and vitK2, in the potential preservation of bone health. Although promising and intriguing, these findings should be interpreted with caution since they are still preliminary and require further elucidation with other well-designed studies.

## Figures and Tables

**Table 1:** Baseline characteristics of all participants.

Baseline Characteristics	All (*n* = 45)	Group A (*n* = 22)	Group B (*n* = 23)	*p*-Value
Age (years)	53 (49–56)	52.50 (49.00–56.75)	53 (49.50–55)	0.900
Height (cm)	166 (162–168)	165 (162–166.75)	166 (165–168)	0.133
Weight (kg)	66 (62–73)	65.5 (60.25–72.50)	66 (65–74)	0.304
BMI (kg/m^2^)	23.38 (22.49–27)	23.19 (22.225–26.54)	23.4 (22.665–27.00)	0.810
Age at menopause (years)	50.5 (48.75–52)	50 (48–51)	51 (49–52)	0.451
L1-L4 TBS (unitless/score)	1.314 (1.269–1.358)	1.314 (1.269–1.38)	1.299 (1.282–1.348)	0.893
L1-L4 BMD (g/cm^2^)	0.966 (0.88–1.05)	0.96 (0.878–1.033)	0.966 (0.881–1.05)	0.554
Total hip BMD (g/cm^2^)	0.839 (0.762–0.864)	0.84 (0.776–0.867)	0.838 (0.752–0.86)	0.927
Femoral neck BMD (g/cm^2^)	0.785 (0.73–0.84)	0.753 (0.72–0.805)	0.786 (0.733–0.849)	0.832
L1-L4 T-score (unitless/SD)	−1 (−1.9–−0.3)	−0.95 (−1.85–−0.325)	−1 (−2.05–−0.25)	0.927
Parathyroid hormone (PTH) (pg/mL)	57 (41.75–63.4)	55 (42–64.6)	58 (41–62.5)	1.000
Phosphorus (mg/dL)	3.1 (2.9–3.7)	3 (2.9–3.6)	3.24 (2.9–3.77)	0.419
Calcium (mg/dL)	9.4 (8.9–9.9)	9.4 (8.9–9.9)	9.3 (8.9–9.82)	0.902
Vitamin D (ng/mL)	22.7 (17–29)	22.7 (17–28)	22 (17–28.25)	0.934
*C*-terminal telopeptide (CTX) (ng/mL)	0.77 (0.42–0.9)	0.64 (0.42–0.89)	0.78 (0.42–0.9)	0.801
Osteocalcin (ng/mL)	20.5 (18–24)	21.5 (19.25–24)	20 (16.85–23)	0.265
FSH (mIU/mL)	77 (57.43–88)	72.8 (56.75–88.5)	77 (59.81–87.6)	0.930
Estradiol (pg/mL)	18.4 (10–20)	18.7 (10–30.4)	18.4 (10–20)	0.528
Vasomotor domain (score)	5.66 (4–7.33)	6.3 (4.33–7.33)	4.33 (2.83–7)	0.06
Psychosocial domain (score)	5 (2.57–6.14)	5.42 (4.28–6.25)	4.14 (2.42–5.64)	0.122
Physical domain (score)	4.06 (3.12–4.93)	4.5 (3.34–5.12)	3.75 (3.09–4.65)	0.351
Sexual domain (score)	4.66 (2.3–6.6)	4.5 (2.3–6.9)	5 (2.3–6)	0.656

Data are shown as median and interquartile ranges. Comparisons between the supplement and control groups were performed using a Mann–Whitney U-test. A *p*-value greater than 0.05 indicates that there are no statistically significant differences between the two groups at baseline (T0).

**Table 2:** Comparison of changes in serum biochemical parameters and MENQOL domains between the treatment and control groups after 3 months.

	Baseline	Follow-Up	Treatment Effect	FDR-Adjusted Within-Group and Between-Group *p*-Values (Effect Size)	
Parathyroid hormone (PTH)					
Group A	55 (42–64.6)	55 (51–59.67)	−1.85 (−13–10)	0.5685 (−0.04)	0.2946 (0.16)
Group B	58 (42–62.5)	60.5 (51–64.9)	0 (−9.2–0.5)	0.2156 (−0.58)	
Phosphorus					
Group A	3 (2.9–3.6)	3.15 (3–3.5)	−0.1 (−0.3–0.17)	0.4926 (−0.12)	**0.0458 (−0.37)**
Group B	3.24 (2.9–3.77)	3 (2.92–3.1)	0 (0–0.7)	0.2144 (0.76)	
Calcium					
Group A	9.4 (8.9–9.9)	9.55 (9.1–9.87)	−0.2 (−0.8–0.275)	0.2817 (−0.25)	0.5223 (0.04)
Group B	9.3 (8.9–9.82)	9.6 (9.12–9.9)	0 (−0.4–0)	0.3978 (−0.56)	
Vitamin D					
Group A	22.7 (16.25–28)	37 (30.8–41)	−10.3 (−21–−2.47)	**0.0004 (−0.94)**	**0.0002 (−0.69)**
Group B	22 (17–28.25)	17 (23.75–30.85)	0 (−2.5–10.65)	0.4336 (0.36)	
*C*-terminal telopeptide (CTX)					
Group A	0.64 (0.42–0.89)	0.44 (0.34–0.7)	0.12 (−0.05–0.33)	**0.0415 (0.56)**	**0.0458 (0.37)**
Group B	0.78 (0.42–0.9)	0.88 (0.34–0.96)	0 (−0.47–0.00)	0.8005 (−0.21)	
Osteocalcin					
Group A	21.5 (24–19.25)	20 (17.5–22)	0.5 (−3.15–8.5)	0.5685 (0.06)	0.3966 (0.13)
Group B	20 (19.25–23)	21 (17.75–23)	0 (−7.8–5)	1.0000 (0.05)	
Vasomotor domain					
Group A	6.3 (4.33–7.33)	2.66 (1–3.66)	3.33 (2–4.33)	**0.0004 (0.95)**	**0.0001 (0.76)**
Group B	4.33 (4.33–7)	4.66 (1.16–7)	0 (−0.33–0)	1.0000 (−0.09)	
Psychosocial domain					
Group A	5.18 (2.81–6.34)	2.87 (2.5–3.34)	2.31 (0.34–3.31)	**0.0006 (0.90)**	**0.0008 (0.60)**
Group B	4.25 (4.18–5.56)	4.5 (2.5–5.75)	0 (−0.5–0)	0.4336 (−0.39)	
Physical domain					
Group A	4.5 (3.34–5.12)	2.68 (2–3.23)	1.71 (0.84–2.45)	**0.0004 (0.90)**	**0.0002 (0.70)**
Group B	3.75 (3.34–4.65)	3.93 (2.01–4.75)	−0.12 (0.46–0)	0.4336 (−0.34)	
Sexual domain					
Group A	4.5 (2.3–6.9)	4 (1.3–5)	1 (0–2.41)	0.0706 (0.49)	0.1333 (0.25)
Group B	5.0 (2.66–6.0)	5.33 (1.4–6.5)	0 (−0.16–0.66)	1.0000 (0.00)	

Data are expressed as median and interquartile range (IQR: 1st–3rd quartile). Group A (treated) received a combination treatment with Purified Cytoplasm of Pollen (PCP), vitamin D3, and vitamin K2; Group B (control) received no active treatment. The “Treatment Effect” column represents the median of the individual changes calculated as Δ = T0 − T3. The *p*-values reported in the final section refer to the FDR-adjusted within-group changes (calculated using the Wilcoxon signed-rank test) and the between-group comparison of the treatment effect or delta (calculated using the Wilcoxon rank-sum test). Statistically significant *p*-values are highlighted in bold.

## Data Availability

The raw data supporting the conclusions of this article will be made available by the authors on request. The data are not publicly available due to privacy.

## Supplementary Materials

The following supporting information can be downloaded at https://www.mdpi.com/article/10.3390/nu18162734/s1: Figure S1: Strobe flow chart.

## Appendix A

**Table A1:** Table showing the *p*-values for individual MenQOL items.

Item	Baseline	Follow-Up	Treatment Effect	Intra-Group *p*-Value	Inter-Group *p*-Value
**Vasomotor Domain**					
Hot flashes					
Group A	6 (4.25–8)	1 (1–4)	3.5 (1–5)	**0.0004**	**0.0001**
Group B	5 (2–6.5)	5 (2–7)	0 (−1–0)	1.0000	
Night sweats					
Group A	7 (6–8)	3 (1–4)	3.5 (2–5)	**0.0004**	**0.0005**
Group B	6 (3.5–7)	6 (2–7)	0 (0–0)	1.0000	
Sweating					
Group A	7 (5–7)	3 (1–4)	3 (1.25–4.75)	**0.0012**	**0.0006**
Group B	5 (1–7)	5 (2–7)	0 (−0.5–0)	1.0000	
**Psychosocial Domain**					
Being dissatisfied with my personal life					
Group A	5 (1–7)	4 (1–4)	0.5 (−1.75–2.75)	0.0830	0.0625
Group B	1 (1–6)	3 (1–6)	0 (0–0)	1.0000	
Feeling anxious or nervous					
Group A	6.5 (4.25–7)	3 (1–4)	2.5 (1–4)	**0.0004**	**0.0001**
Group B	6 (5.5–7)	6 (5–7)	0 (−0.5–0)	1.0000	
Experiencing poor memory					
Group A	6.5 (3.25–7)	3 (1.5–4)	3 (2–3.75)	**0.0010**	**0.0001**
Group B	5 (3–6)	6 (4–6.5)	0 (−1.5–0)	1.0000	
Accomplishing less than I used to					
Group A	2 (1–7)	3 (1–4)	0 (0–3)	0.0691	0.0815
Group B	1 (1–6)	1 (1–6)	0 (0–0)	1.0000	
Feeling depressed, down or blue					
Group A	6 (3.25–7.75)	3 (1–3.75)	3.5 (0.25–4)	**0.0006**	**0.0003**
Group B	3 (1–5)	5 (1–5.5)	0 (0–0)	1.0000	
Being impatient with other people					
Group A	6.5 (4.5–7)	3 (1–5)	3 (0–5)	**0.0083**	**0.0057**
Group B	5 (1–7)	5 (2.5–7)	0 (0–0)	1.0000	
Feeling of wanting to be alone					
Group A	5 (3–7)	1 (1–3.75)	3 (0–4)	**0.0007**	**0.0012**
Group B	4 (1–7)	5 (1–7)	0 (0–0)	1.0000	
**Physical Domain**					
Flatulence					
Group A	2.5 (1–7)	1 (1–2.5)	0 (0−5.75)	**0.0105**	0.0481
Group B	1 (1–6)	1 (1–6.5)	0 (0–0)	1.0000	
Aching in muscles and joints					
Group A	3.5 (1–6)	2.5 (1–4)	0 (−0.75–4)	0.0424	0.1476
Group B	5 (1–6.5)	4 (6.5–1)	0 (0–0)	1.0000	
Feeling tired or worn out					
Group A	6 (3.25–7)	4 (4–4)	2 (0–3)	**0.0186**	**0.0109**
Group B	4 (1–7)	6 (2.5–7)	0 (−0.5–0)	1.0000	
Difficulty sleeping					
Group A	7 (4.25–8)	3 (1–4)	4 (0.25–4.75)	**0.0007**	**0.0007**
Group B	6 (4.5–8)	6 (4.5–8)	0 (−0.5–0)	1.0000	
Aches in back of neck or head					
Group A	5 (1–6)	3 (1–4)	0 (0–3.75)	**0.0141**	**0.0367**
Group B	5 (1–6)	4 (1–7)	0 (0–0)	1.0000	
Decrease in physical strength					
Group A	7 (1.75–8)	3 (1.5–4)	3 (0–4)	**0.0008**	**0.0012**
Group B	4 (1–7)	4 (1–7)	0 (0–0)	1.0000	
Decrease in stamina					
Group A	7 (4–8)	4 (3–4)	3 (0.25–4)	**0.0011**	**0.0008**
Group B	4 (1–7)	5 (1–7)	0 (−0.5–0)	1.0000	
Feeling a lack of energy					
Group A	7 (4.25–7)	3.5 (3–4)	3 (1–4)	**0.0035**	**0.0006**
Group B	6 (3.5–7)	6 (4–7)	0 (0–0)	1.0000	
Drying skin					
Group A	1 (1–4.75)	1 (1–1)	0 (0–2.75)	**0.0071**	**0.0017**
Group B	4 (1–6)	4 (1–6)	0 (−2–0)	1.0000	
Weight gain					
Group A	5.5 (5–7.75)	4 (3–5)	1 (0–3.75)	**0.0034**	**0.0085**
Group B	6 (4–7)	6 (4.5–7)	0 (0–0)	1.0000	
Increased facial hair					
Group A	1 (1–1)	1 (1–1)	0 (0–0)	0.2178	0.1484
Group B	1 (1–1)	1 (1–1)	0 (0–0)	1.0000	
Changes in appearance, texture or tone of your skin					
Group A	1 (1–5.75)	1 (1–4)	0 (0–1)	**0.0341**	**0.0232**
Group B	4 (1–6)	4 (1–6)	0 (−1.5–0)	1.0000	
Feeling bloated					
Group A	7 (3.5–7)	3 (3–4)	2.5 (0.25–4)	**0.0035**	**0.0033**
Group B	6 (4–7)	6 (4.5–7)	0 (−1–0)	1.0000	
Low backache					
Group A	4.5 (1–6)	3 (1–4)	1.5 (0–3)	**0.0203**	0.0852
Group B	1 (1–5)	1 (1–5)	0 (0–0)	1.0000	
Frequent urination					
Group A	1 (1–6.75)	1 (1–2.5)	0 (0–4)	**0.0151**	**0.0163**
Group B	1 (1–5)	2 (1–5)	0 (0–0)	1.0000	
Involuntary urination when laughing or coughing					
Group A	1 (1–4.5)	1 (1–1)	0 (0–1.75)	**0.0093**	**0.0110**
Group B	1 (1–5)	1 (1–5)	0 (0–0)	1.0000	
**Sexual Domain**					
Change in your sexual desire					
Group A	5 (4–6.75)	4 (1.5–5)	1 (0–3)	**0.0083**	**0.0273**
Group B	5 (3.5–7)	6 (4.5–7)	0 (0–0)	1.0000	
Vaginal dryness during intercourse					
Group A	5 (1–7)	4.5 (1–6)	0 (0–2.75)	0.1342	0.1412
Group B	4 (1–7)	4 (1–6.5)	0 (0–0)	1.0000	
Avoiding intimacy					
Group A	5 (1–7)	4 (1–5)	0.5 (0–1.75)	**0.0297**	**0.0235**
Group B	5 (1–6)	5 (2–7)	0 (−1–0)	1.0000	

Scores and statistical analysis of individual MenQOL items. Data are expressed as median and interquartile range (IQR: 1st–3rd quartile). The table reports baseline and follow-up values, along with the treatment effect, for both the treated group (Group A) and the control group (Group B). Statistically significant *p*-values (*p* < 0.05) are highlighted in bold.

## References

[1] Vaccaro C.M., Capozzi A., Ettore G., Bernorio R., Cagnacci A., Gambacciani M., Coletta V., Maffei S., Nappi R.E., Scambia G. (2021). What women think about menopause: An Italian survey. Maturitas.

[2] Gast G.C., Grobbee D.E., Pop V.J., Keyzer J.J., Wijnands-van Gent C.J., Samsioe G.N., Nilsson P.M., van der Schouw Y.T. (2009). Vasomotor symptoms are associated with a lower bone mineral density. Menopause.

[3] The NAMS 2017 Hormone Therapy Position Statement Advisory Panel (2017). The 2017 hormone therapy position statement of The North American Menopause Society. Menopause.

[4] Cagnacci A., Gambacciani M., Gallo M., Lello S. (2019). Executive Committee of the Italian Society of Menopause (SIM) and of the Italian Society of Gynaecology of the Third Age (SIGiTE). Recommendations on menopausal hormone replacement therapy. Minerva Ginecol..

[5] Shufelt C.L., Brown V., Carpenter J.S., Chism L.A., Faubion S.S., Joffe H., Kling J.M., Soares C.N., Thurston R.C. (2023). The 2023 nonhormonal therapy position statement of The North American Menopause Society. Menopause.

[6] Cagnacci A., Volpe A., Di Carlo C., De Leo V., Bifulco G., Gambacciani M., Alfieri S., Biglia N., Bonaccorsi G., Caruso S. (2021). Phytotherapy for menopausal symptoms: Recommendations of the Italian menopause society (SIM) and the Italian society of gynaecology for the third age (SIGiTE). Ital. J. Gynaecol. Obstet..

[7] Aarshageetha P., Janci P.R.R., Tharani N.D. (2023). Role of Alternate Therapies to Improve the Quality of Life in Menopausal Women: A Systematic Review. J. Midlife Health.

[8] Pérez-López F.R., Brincat M., Erel C.T., Tremollieres F., Gambacciani M., Lambrinoudaki I., Moen M.H., Schenck-Gustafsson K., Vujovic S., Rozenberg S. (2012). EMAS position statement: Vitamin D and postmenopausal health. Maturitas.

[9] Nuti R., Gennari L., Cavati G., Caffarelli C., Frediani B., Gonnelli S., Laurentaci C., Mauro G.L., Malavolta N., Minisola G. (2025). Vitamin D intake in Italian healthy subjects and patients with different pathological disorders. Front. Nutr..

[10] Nesby-O’Dell S., Scanlon K.S., Cogswell M.E., Gillespie C., Hollis B.W., Looker A.C., Allen C., Doughertly C., Gunter E.W., Bowman B.A. (2002). Hypovitaminosis D prevalence and determinants among African American and white women of reproductive age: Third National Health and Nutrition Examination Survey, 1988–1994. Am. J. Clin. Nutr..

[11] Holick M.F., Binkley N.C., Bischoff-Ferrari H.A., Gordon C.M., Hanley D.A., Heaney R.P., Murad M.H., Weaver C.M. (2011). Endocrine Society. Evaluation, treatment, and prevention of vitamin D deficiency: An Endocrine Society clinical practice guideline. J. Clin. Endocrinol. Metab..

[12] Hilditch J.R., Lewis J., Peter A., Van Maris B., Ross A., Franssen E., Guyatt G.H., Norton P.G., Dunn E. (1996). A menopause specific quality of life questionnaire: Development and psychometric properties. Maturitas.

[13] Ahmed H.S. (2026). Vitamin D and Mineral Ion Regulation. Adv. Exp. Med. Biol..

[14] Wimalawansa S.J., Weiss S.T., Hollis B.W. (2024). Integrating Endocrine, Genomic, and Extra-Skeletal Benefits of Vitamin D into National and Regional Clinical Guidelines. Nutrients.

[15] Parveen B., Parveen A., Vohora D. (2019). Biomarkers of Osteoporosis: An Update. Endocr. Metab. Immune Disord.-Drug Targets.

[16] Hoong C.W.S., Saul D., Khosla S., Sfeir J.G. (2025). Advances in the management of osteoporosis. BMJ.

[17] Bhattoa H.P., Vasikaran S., Trifonidi I., Kapoula G., Lombardi G., Jørgensen N.R., Pikner R., Miura M., Chapurlat R., Hiligsmann M. (2025). Update on the role of bone turnover markers in the diagnosis and management of osteoporosis: A consensus paper from The European Society for Clinical and Economic Aspects of Osteoporosis, Osteoarthritis and Musculoskeletal Diseases (ESCEO), International Osteoporosis Foundation (IOF), and International Federation of Clinical Chemistry and Laboratory Medicine (IFCC). Osteoporos. Int..

[18] Topyürek D., Meier C., Kränzlin M.E. (2025). Der Stellenwert der Knochenumbauparameter im Management der Osteoporose [The importance of bone remodelling parameters in the management of osteoporosis]. Ther. Umsch..

[19] Elahmer N.R., Wong S.K., Mohamed N., Alias E., Chin K.Y., Muhammad N. (2024). Mechanistic Insights and Therapeutic Strategies in Osteoporosis: A Comprehensive Review. Biomedicines.

[20] Capozzi A., Scambia G., Migliaccio S., Lello S. (2020). Role of vitamin K2 in bone metabolism: A point of view and a short reappraisal of the literature. Gynecol. Endocrinol..

[21] Jadhav N., Ajgaonkar S., Saha P., Gurav P., Pandey A., Basudkar V., Gada Y., Panda S., Jadhav S., Mehta D. (2022). Molecular Pathways and Roles for Vitamin K2-7 as a Health-Beneficial Nutraceutical: Challenges and Opportunities. Front. Pharmacol..

[22] Gibson C.J., Thurston R.C., Matthews K.A. (2016). Cortisol dysregulation is associated with daily diary-reported hot flashes among midlife women. Clin. Endocrinol..

[23] Reed S.D., Newton K.M., Larson J.C., Booth-LaForce C., Woods N.F., Landis C.A., Tolentino E., Carpenter J.S., Freeman E.W., Joffe H. (2016). Daily salivary cortisol patterns in midlife women with hot flashes. Clin. Endocrinol..

[24] Genazzani A., Panay N., Simoncini T., Depypere H., Mueck A., Egarter C., Biglia N., Fait T., Birkhaeuser M., Skouby S.O. (2020). Purified and specific cytoplasmic pollen extract: A non-hormonal alternative for the treatment of menopausal symptoms. Gynecol. Endocrinol..

[25] Lello S., Capozzi A., Xholli A., Cagnacci A. (2022). The benefits of purified cytoplasm of pollen in reducing menopausal symptoms in peri- and post-menopause: An Italian multicenter prospective observational study. Minerva Obstet. Gynecol..

[26] De Franciscis P., Conte A., Schiattarella A., Riemma G., Cobellis L., Colacurci N. (2020). Non-hormonal Treatments for Menopausal Symptoms and Sleep Disturbances: A Comparison Between Purified Pollen Extracts and Soy Isoflavones. Curr. Pharm. Des..

[27] Lello S., Paris I., Cagnacci A., Sartori D., Caruso S., Iop A. (2023). Vasomotor symptoms and management of women undergoing treatment for breast cancer: Literature review with focus on the therapeutic potential of cytoplasmic pollen extract. Gynecol. Endocrinol..

[28] Bounous V.E., Cipullo I., D’Alonzo M., Martella S., Franchi D., Villa P., Biglia N., Ferrero A. (2024). A prospective, multicenter, randomized, double-blind placebo-controlled trial on purified and specific Cytoplasmic pollen extract for hot flashes in breast cancer survivors. Gynecol. Endocrinol..

[29] Appel K., Chedraui P., Nappi R.E., Palacios S., Genazzani A.R., Simoncini T. (2025). The in vitro effects of purified and specific cytoplasmic pollen extract on dopaminergic and GABAergic systems. Gynecol. Endocrinol..

[30] Winther K., Rein E., Hedman C. (2005). Femal, a herbal remedy made from pollen extracts, reduces hot flushes and improves quality of life in menopausal women: A randomized, placebo-controlled, parallel study. Climacteric.

[31] Agarwal K., Nagendra L., Bhattacharya S. (2025). Approach to premenopausal osteoporosis. Curr. Opin. Endocrinol. Diabetes Obes..

[32] Grewal J., Sowers M.R., Randolph J.F., Harlow S.D., Lin X. (2006). Low bone mineral density in the early menopausal transition: Role for ovulatory function. J. Clin. Endocrinol. Metab..

[33] Acquarulo E.L., Hernandez E.C., Kodzodziku F., Nemec E.C. (2024). The efficacy of purified pollen extract for reducing vasomotor symptoms in women: A systematic review and meta-analysis. Menopause.

