# Supplementary material for: Beneficial Effects of Purified Cytoplasm of Pollen Associated with Vitamin D and Vitamin K2 on Menopausal Symptoms and Bone Health
Source: Nutrients. 2026 Aug 21;18(16):2734. doi: 10.3390/nu18162734 (PMC13516513; doi:10.3390/nu18162734)

Strobe flow-chart

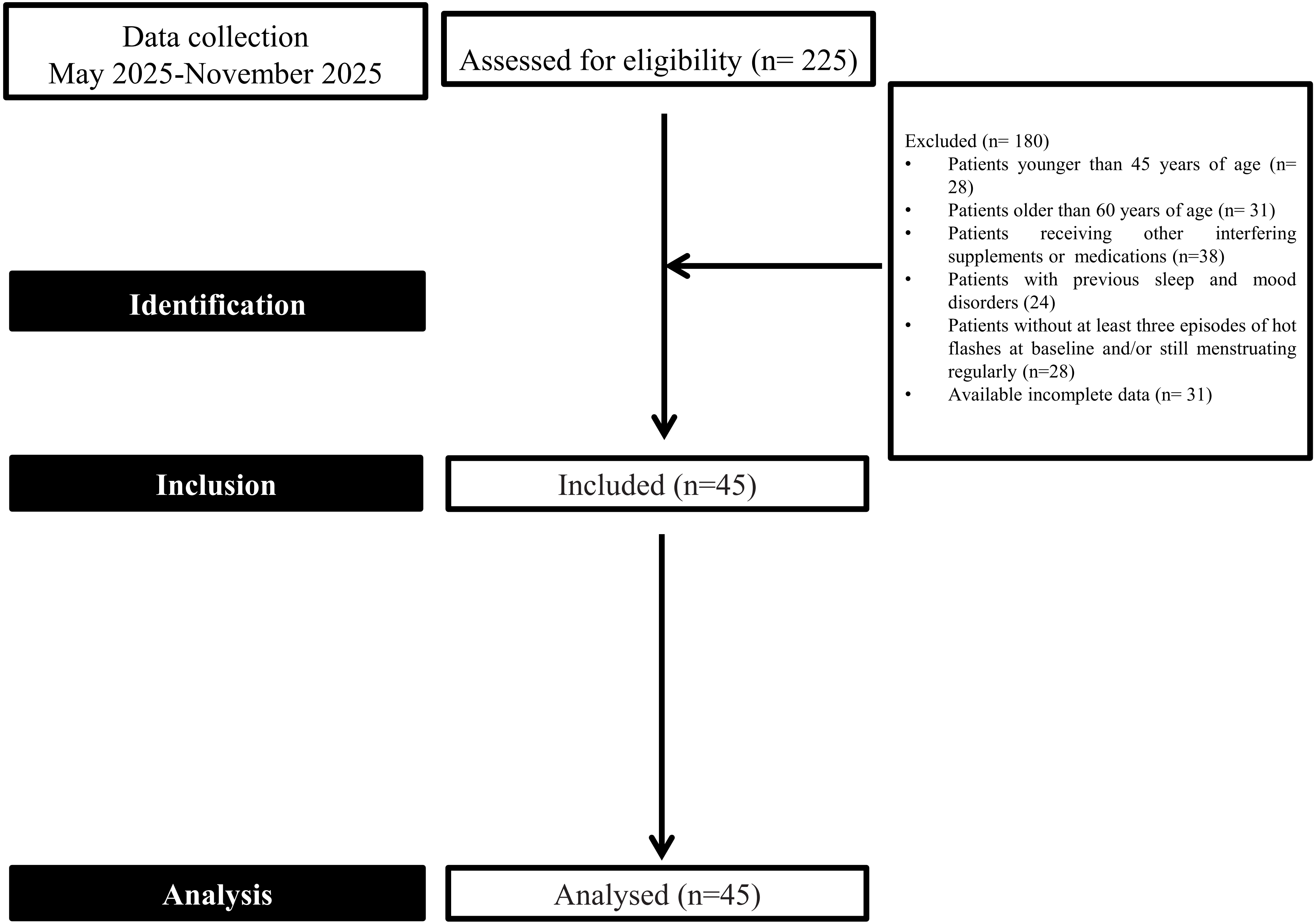

Supplement: Supplementary file 1 [file nutrients-18-02734-s001.zip › nutrients-4468179-supplementary.pdf]
